# Implementation Science to Accelerate Clean Cooking for Public Health

**DOI:** 10.1289/EHP1018

**Published:** 2017-01-01

**Authors:** Joshua Rosenthal, Kalpana Balakrishnan, Nigel Bruce, David Chambers, Jay Graham, Darby Jack, Lydia Kline, Omar Masera, Sumi Mehta, Ilse Ruiz Mercado, Gila Neta, Subhrendu Pattanayak, Elisa Puzzolo, Helen Petach, Antonello Punturieri, Adolfo Rubinstein, Michael Sage, Rachel Sturke, Anita Shankar, Kenny Sherr, Kirk Smith, Gautam Yadama

**Affiliations:** 1 Division of Epidemiology and Population Studies, Fogarty International Center, National Institutes of Health, Bethesda, Maryland, USA; 2 Department of Environmental Health Engineering, Sri Ramachandra University, Chennai, India; 3 Department of Public Health and Policy, University of Liverpool, Liverpool, United Kingdom; 4 National Cancer Institute, National Institutes of Health, Rockville, Maryland, USA; 5 Department of Environmental and Occupational Health, George Washington University, Washington, DC, USA; 6 Department of Environmental Health Sciences, Columbia University, New York, New York, USA; 7 Instituto de Investigaciones en Ecosistemas, National Autonomous University of Mexico, Morelia, Mexico; 8 Global Alliance for Clean Cookstoves, Washington, DC, USA; 9 Sanford School of Public Policy & Nicholas School of the Environment, Duke University, Durham, North Carolina, USA; 10 Global LPG Partnership, New York, New York, USA; 11 U.S. Agency for International Development, Washington, DC, USA; 12 National Heart, Lung, and Blood Institute, Rockville, Maryland, USA; 13 Institute for Clinical Health Effectiveness, Buenos Aires, Argentina; 14 Centers for Disease Control and Prevention, Atlanta, Georgia, USA; 15 Department of Environmental Health Sciences, Johns Hopkins University, Baltimore, Maryland, USA; 16 Department of Global Health, University of Washington, Seattle, Washington, USA; 17 Department of Global Environmental Health, University of California, Berkeley, California, USA; 18 George Warren Brown School of Social Work, Washington University, St. Louis, Missouri, USA

## Abstract

Clean cooking has emerged as a major concern for global health and development because of the enormous burden of disease caused by traditional cookstoves and fires. The World Health Organization has developed new indoor air quality guidelines that few homes will be able to achieve without replacing traditional methods with modern clean cooking technologies, including fuels and stoves. However, decades of experience with improved stove programs indicate that the challenge of modernizing cooking in impoverished communities includes a complex, multi-sectoral set of problems that require implementation research. The National Institutes of Health, in partnership with several government agencies and the Global Alliance for Clean Cookstoves, has launched the Clean Cooking Implementation Science Network that aims to address this issue. In this article, our focus is on building a knowledge base to accelerate scale-up and sustained use of the cleanest technologies in low- and middle-income countries. Implementation science provides a variety of analytical and planning tools to enhance effectiveness of clinical and public health interventions. These tools are being integrated with a growing body of knowledge and new research projects to yield new methods, consensus tools, and an evidence base to accelerate improvements in health promised by the renewed agenda of clean cooking.

## Introduction

Three billion people cook with traditional biomass stoves and open fires. Results from the Global Burden of Disease (GBD) Study have estimated that the consequent household air pollution (HAP) causes almost four million premature deaths annually—a health burden borne largely by the poor, women, and children in low-income countries (Lim et al. 2012). HAP has been linked both to some of the major preventable causes of death in children (including low birth weight and respiratory infections), and to some of the most significant contributors of non-communicable diseases (NCDs), morbidity, and mortality around the world: stroke, CVD, chronic pulmonary disease, lung cancer and eye disease, as well as several safety concerns such as burns, injuries, and gender-based violence associated with biomass collection and use (Lim et al. 2012).

The challenge is to accelerate the widespread, sustained adoption of demonstrably clean cooking to promote public health. Implementation science has evolved to meet precisely this kind of complex, multidimensional challenge. Both the scale and the complexity of implementing cleaner household energy practices in low- and middle-income countries (LMICs) call for systematic attention, not only to supply costs and cooking behavior by (largely) the women in the household, but also to the household, community, and larger socioeconomic and environmental context of energy access, adoption, and use (Jeuland and Pattanayak 2012). Actions by multiple sectors (e.g., energy, banking, communication, and commercial services), beyond health, are needed. Poverty, access to services, home construction, climate, cultural traditions, gender differences in opportunity costs, and time preferences are just some of the persistent barriers to be addressed.

In 2014, the World Health Organization (WHO) released its indoor air quality guidelines (IAQG) for household fuel combustion (WHO 2014). The guidelines were developed to address the public health burden from household air pollution. Development of these guidelines began with the previously published WHO air quality guideline values for specific pollutants, including for fine particulate matter (PM_2.5_) and carbon monoxide (WHO 2006, 2010) that drew on reviews of a wide range of evidence spanning fuel use, emission levels and testing, exposure levels around the world, health risks, impacts of interventions in everyday use on HAP, and factors influencing sustained adoption.

One of the key conclusions from the IAQG was that, despite impressive exposure reductions of 50–80% in the best stove programs, in absolute terms average post-intervention concentrations remained well above the WHO interim target (35 μg/m^3^ annual mean)—that is, levels estimated to be necessary to yield significant health improvements (WHO 2014). Based on the limited data available at the time, clean fuel technologies [e.g., liquid petroleum gas (LPG), biogas, electricity, ethanol] performed best overall, but households using them also fell short of the target. Stove stacking (using multiple stoves and fuels) and other pollution sources inside (e.g., kerosene lamps) and outside the home were likely explanations. These findings suggest that near exclusive, community-wide use of clean fuels is needed to meet the PM_2.5_ guideline and to maximize health benefits (Johnson and Chiang 2015).

Most of the developed world, as well as the high-income populations in the developing world, have made the transition to cleaner fuels for cooking and other household energy needs (IEA 2015). While next generation solid fuel stoves may provide an important transitional technology with potential health benefits, this has yet to be demonstrated, and a recent study highlights the emission challenges these technologies face (Mutlu et al. 2016). Therefore, the challenge before the public health community is how to accelerate the movement of large numbers of lower-income people up the energy ladder to cleaner cooking, while recognizing that the transition to clean fuels will vary according to socioeconomic status and geography (Jeuland et al. 2015). In addition, stove and fuel stacking will continue to occur for a variety of reasons (Ruiz-Mercado and Masera 2015).

## Methods

Recently, several systematic reviews have been published that focused on the factors that influence successful adoption and sustained use of clean cooking and household energy projects around the world (Jeuland and Pattanayak 2012; Puzzolo et al. 2013, 2016; Rehfuess et al. 2014). Puzzolo et al. (2013, 2016) identified 31 factors grouped under seven domains that we take as a starting point. In the following list, we outline these domains and provide examples of key considerations for researchers and implementers regarding enablers and barriers to clean cooking (adapted from Puzzolo et al. 2013, 2016).

*Fuel and technology characteristics*. Has the technology proven to be clean enough in realistic use cases and for the main cooking tasks? Is it physically capable of meeting local needs for ease of use and cooking traditions? Does it require high levels of maintenance? Does it reduce total time and monetary expenditures associated with cooking?
*Household and setting characteristics*. Will the new household energy technology be used indoors, outdoors or both? What complementary technologies, practices and structural changes can minimize stacking and its impact on health? Will other energy needs, such as heating, lighting, or drying food be a significant barrier to reducing HAP?
*Knowledge and perceptions*: Does the technology address social status and safety concerns? Do users receive adequate information to operate and maintain the technology? Do both women and men see clean cooking as desirable? Note that women are often the primary users of cooking technology and frequently suffer disproportionately from HAP, yet men tend to decide on technology investments.
*Financial, tax and subsidy aspects.* Is the stove technology, including needed fuels and maintenance costs, affordable to the end user now, or will additional subsidies or credit systems to finance sustained use be required? Are plans and resources for these additional costs available and sustainable?
*Market development.* Is the supply chain viable and reliable in the region? Are there effective sales and distribution channels for clean stoves, fuel, and replacement parts, and are these available throughout the year.
*Regulation, legislation and standard*. Are there plans for national regulations that support the WHO guidelines for indoor air quality? Are the ISO clean cookstove standards or a comparable system recognized? Are there guidelines for implementation and strategies for monitoring progress, safety, health and other outcomes?
*Programmatic and policy mechanisms*. Is the broader policy context reasonably aligned with the goals of the cookstove program, or is there government policy that stands opposed to the aims of the program? Are conditions in place to attract the development and commercial investment required to expand access to clean cooking?

These questions are illustrative, rather than comprehensive, and demonstrate why formal system-wide planning is important for both initial implementation and sustainability of an intervention.

The field of implementation science (Glasgow et al. 1999; Madon et al. 2007; Tabak et al. 2012; Yamey 2011) offers an important approach to these issues and may help accelerate scale-up of the most promising technologies and approaches to national and regional challenges (Milat et al. 2015; Yamey 2011). In the remainder of this article, we outline an implementation science approach to accelerate this transition.

Because adoption and use challenges are so central to understanding how clean cooking can provide positive health effects, the Fogarty International Center at the National Institutes of Health (NIH), in partnership with the NIH Common Fund, other NIH partners, the U.S. Agency for International Development, the Centers for Disease Control and Prevention, the U.S. Environmental Protection Agency (EPA), and the Global Alliance for Clean Cookstoves (GACC), has developed a Clean Cooking Implementation Science Network (ISN) (Fogarty International Center 2015). The Clean Cooking ISN brings together leading HAP researchers along with experts in anthropology, economics, rural energy, and implementation science, as well as policy makers and implementers in the HAP arena. Our aim is to develop a comprehensive knowledge base to improve the provision, uptake, and the appropriate and sustained use of evidence-based clean cooking interventions to maximize public health and quality-of-life benefits in LMICs. Over the next five years, we will synthesize and adapt analytical tools for planning and evaluating household energy interventions, develop case studies, support strategically designed and chosen studies to answer key questions, and disseminate our findings and other products as widely as possible to enhance the knowledge base and policy tool kit for scaling up of clean cooking systems around the world.

The Clean Cooking ISN aims to shed light on such questions as:

How we can implement clean, efficient, safe, and resilient cooking systems for the 2.7 billion people that currently rely on traditional stoves, coal, and kerosene for cooking?
What level of clean technology adoption (at both household and community scale) is required to deliver the health benefits implicit in the GBD estimates?
How do households make decisions about meeting their cooking energy needs, and how can insights into this decision process inform policy and program implementation?
What policy and program options should governments and other proponents of clean cooking consider to improve access, affordability, uptake, and maintenance among the poorest populations?
What dissemination and implementation methods as well as monitoring and evaluation tools and technologies enable ongoing learning to support sustained success of HAP interventions?
How can industry and market factors (fuel producers, distributors, and regulators) that affect sustained clean fuels adoption be influenced constructively?
How can the scientific community ensure that clean fuels and clean cooking programs around the world are evaluated in a manner that allows a meaningful comparison of approaches and outcomes?

Current research on the adoption of clean cooking solutions to HAP is highly varied and fragmented in approach (Jeuland and Pattanayak 2012): idiosyncratic methods, contexts, and technologies exacerbate the difficulty of translating results into action. Implementation science has expanded rapidly as a research discipline in the past 15 years and is expressly focused on understanding and supporting the adoption, implementation, and sustainability of effective interventions in clinical and community settings. It provides methods and strategies to translate research findings from diverse interventions into policy and practice to help bridge the gap between what is known and what is actually done, and seeks to understand the behavior of relevant stakeholders such as providers, end users, patients, organizations, and policy makers in a particular context, as key variables to promote uptake.

Unlike interventions tested in most randomized clinical trials, which evaluate the efficacy of interventions on individuals, implementation science seeks to understand the implementation and scale up of proven interventions in complex real world environments. These may include strategies that aim to promote behavior change of producers or providers, or at higher levels such as governments, communities, health systems, or actors outside the health sector (Craig et al. 2013), including for example, those in the clean fuels delivery chain. Moreover, the implementation process may be compromised by problems of acceptability, compliance, delivery of the intervention, recruitment and retention, and smaller than expected effect sizes that could have been prevented if a feasibility or pilot study were planned *ex ante* (Craig et al. 2008).

For these reasons, it is critical that evaluation of complex interventions should include process as well as outcome measures in order to understand the ways in which interventions have been actually implemented. This approach can disentangle components of the intervention and provide valuable insights into why an intervention did not work or had unexpected results, and when it does work, why it did and how it could be optimized. Process evaluations nested within implementation protocols can be used to assess fidelity and quality of implementation, clarify causal mechanisms, and identify contextual factors associated with variation in outcomes (Oakley et al. 2006). Evaluation of process is not a substitute for evaluation of outcomes, but it can be extremely helpful, particularly in studies with null findings, when a complex intervention can be like a black box, with uninterpretable outputs and outcomes (Rubinstein et al. 2015).

## Results

Implementation science approaches provide a variety of systematic analytical frameworks that support planning and prediction, and facilitate experimentation and iterative learning (Nilsen 2015). Further, by examining the process of intervention, dissemination, and implementation through generalized models, researchers and practitioners can utilize a consistency of language across programs to facilitate communication among researchers, implementers, and decision makers.

Among the multitude of implementation science frameworks that have been described in health and related fields, three have achieved prominence for their comprehensiveness, flexibility, and usability and have significant potential for clean cooking interventions: *a*) diffusion of innovations, *b*) RE-AIM, and *c*) consolidated framework for implementation science:

Diffusion of innovations (Tabak et al. 2012) identifies strategies to increase the speed and effectiveness of innovation transfer to the end user and examines key stages in this adoption process:
Knowledge, persuasion, decision, implementation, and confirmation.
The RE-AIM framework (Glasgow et al. 1999) assesses the potential population-level impact of innovations through five dimensions that include:
Reach, effectiveness, adoption, implementation, and maintenance.
The Consolidated Framework for Implementation Research (CFIR) (Damschroder et al. 2009) seeks to understand how and why interventions work differentially by assessing:
Intervention characteristics, outer setting, inner setting, characteristics of individuals involved, and the implementation process.

In Everett Rogers’ diffusion of innovation theory (Rogers 2003), the rate of adoption is used to categorize end users into groups of innovators (the fastest to adopt), early adopters, early majority, late majority, and laggards (Tabak et al. 2012). Examining the adoption process provides a framework to identify motivating factors at both individual and environmental levels that may influence the decision to adopt a technology or adapt it for better alignment with local needs. The diffusion of innovation framework has been previously used to understand adoption of cookstove innovations (see for example, Clark et al. 2015; Pandey and Yadama 1992). However, its utility for structured or policy-directed programs and interventions may be limited beyond basic acceptance of a technology within a home. Understanding the dynamics of stove stacking and the associated complex patterns of use over time will likely require other approaches.

RE-AIM does not provide any underlying theory of change (King et al. 2010). Rather, it presents a pragmatic model for structuring planning and evaluation that is compatible with clean cooking technology delivery approaches. The specific emphasis on adoption, maintenance and implementation draws evaluative attention by researchers to program implementation factors that are often ignored by scientists.

Like RE-AIM, the consolidated framework for implementation research (CFIR) (Damschroder et al. 2009) is pragmatic and independent of any specific change theory. It provides a comprehensive general structure for unpacking the complex process of real world implementation across multiple settings and can guide formative evaluations of both current and future initiatives to expand the availability and use of cooking technology. The CFIR developers have incorporated the RE-AIM factors but have also allowed for multiple policy environments that influence access to technology in a simple online organizational tool (http://cfirguide.org/).

Furthermore, growing interest in understanding the implementation factors in complex interventions has led to development of hybrid designs for effectiveness and implementation trials (Curran et al. 2012). These may be of particular interest for clean cooking effectiveness trials in which a large number of factors (e.g., fuel supply, price fluctuations, ambient air quality events, home construction) are outside the control of the research team but may be very influential in determining outcomes.

By applying implementation frameworks to studies of adoption of clean cooking, we gain such opportunities as:

Standardize metrics of process, outputs, and outcomes.
Facilitate rigorous planning and evaluation.
Reduce information costs to program entry
Facilitate learning from other fields.

Explicit analytical frameworks also provide structure for designing impact evaluations that apply rigorous measurement and evaluation methodologies to identify interventions that lead to the greatest uptake (e.g., incentives, integrated programming, and optimal delivery models).

### Evaluating the Ecosystem to Support Scaling of Clean Fuels and Technology

In choosing among clean fuels or combinations (e.g., LPG, biogas, alcohol, electricity, solar, tier 4 indoor emissions biomass stoves) and technologies that represent the best option for any given setting, a few “ecosystem” level factors are likely to have a major role (Lewis and Pattanayak 2012; Malla and Timilsina 2014; Puzzolo et al. 2016; Rehfuess et al. 2014; Smith and Sagar 2014). For example, while biogas may be both clean and cost effective in rural areas where dung from large animals is available to provide a substrate for home fuel generation projects, it is unlikely to be practical or scalable in urban and peri-urban settings with currently available methods.

Like some other important household health interventions, such as sanitation and nutrition, changes in energy systems intrinsically must engage with major institutional actors outside the health sector. Thus, major developments in innovation science for HAP alleviation need to bring the energy, investment, and other industries and associated government agencies to the table to apply their skills to the problem, if for no other reason than their activities fundamentally influence access and cost of household energy technologies.

**Figure 1:**
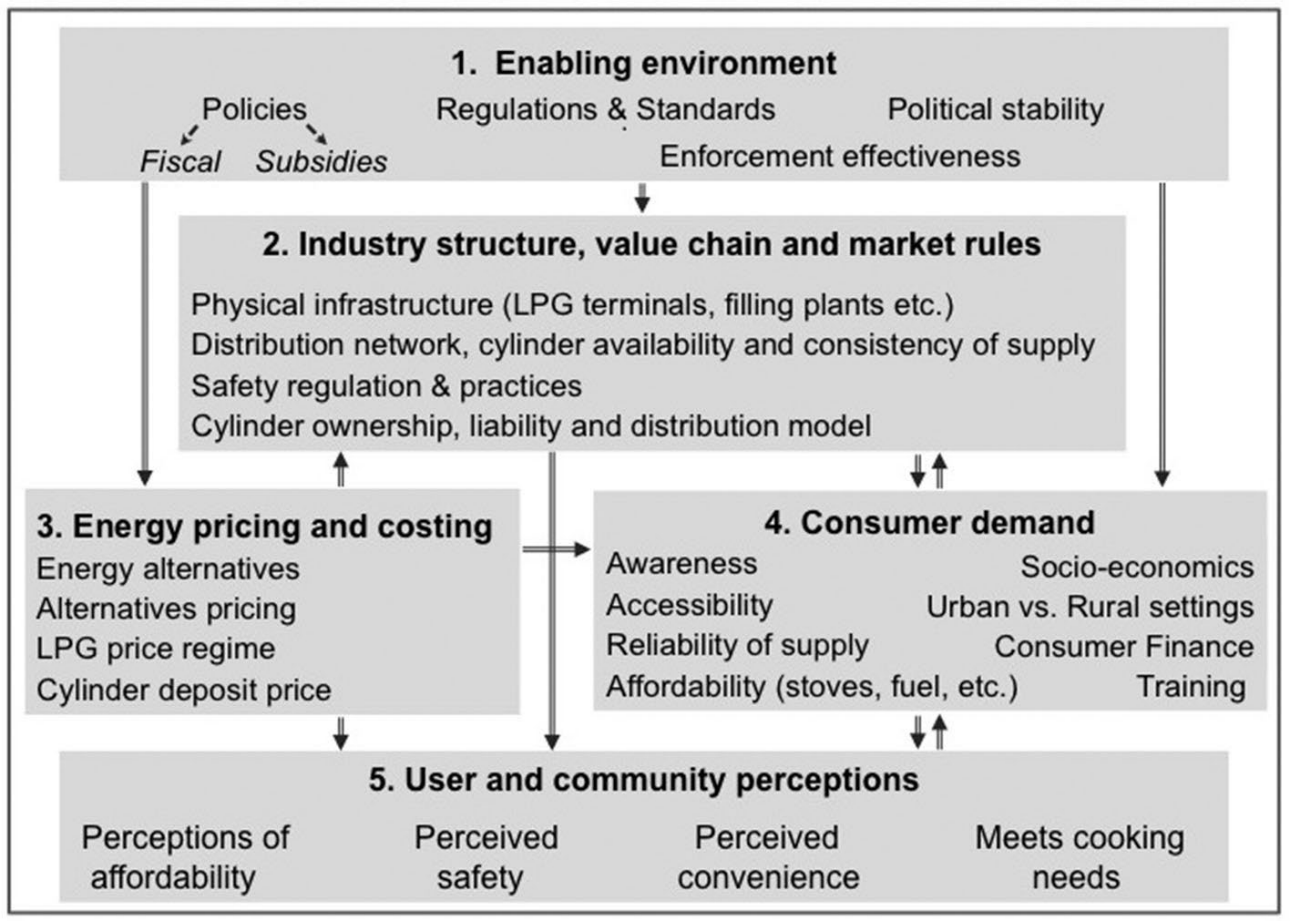
Key dimensions and factors for LPG scaling up and sustained adoption.

In making these assessments, use of a fuel-specific logic model may be helpful to map out basic features and needs of a given technology. For many countries LPG is considered the most practical and scalable clean fuel today for cooking (Malla and Timilsina 2014; Smith and Sagar 2014). Figure 1 illustrates an ecosystem map for this fuel, derived from previous reviews (Puzzolo et al. 2016; WLPGA 2013). Such a model is compatible with the CFIR framework outlined above and its use can, at a minimum, identify information needs early on.

## Discussion

### Evaluating Critical Factors to Maximize Uptake at the Community and Household Level

Surveys, ethnographies and randomized trials are underway around the world to understand a wide variety of influences that affect community and household choices for cooking. These influences include, among others, the role of fuel pricing and capital needs, fuel supply security, gender, background knowledge of health and environmental effects, education and wealth, prestige, time savings, drudgery reduction, and cultural and religious preferences (Jeuland and Pattanayak 2012; Puzzolo et al. 2013; Ruiz-Mercado and Masera 2015; Shankar et al. 2015). The near universal tendencies toward fuel and stove stacking with traditional fires, even when clean technologies are available, is a potent force that requires careful localized understanding to plan for successful interventions and long-term planning to accelerate the transition to communities meeting a progressively higher proportion of energy needs from clean sources (Frangos et al. 2011; Jeuland et al. 2015; Ruiz-Mercado et al. 2011; Ruiz-Mercado and Masera 2015; Yadama 2014).

Community-based system dynamics (Hovmand 2014) is a relatively new approach to understanding the drivers of adoption and sustained use at household and community scale. Systems modeling has been used to understand complex systems in household energy (Howells et al. 2005) and more recently in public health (Homer and Hirsch 2006; Sterman 2006). Community-based systems methods are founded in participatory processes with focus groups or related stakeholder groups (Buchholz et al. 2007; Hovmand 2014; Pandey and Yadama 1992) and variants of this approach are widely used in natural resources management, including water and forestry planning. They are especially valuable in understanding a comprehensive and localized map of influences on socioeconomic choices (Mendoza and Prabhu 2005; Tidwell et al. 2004). More recent application of the method represents an opportunity to both engage communities in the process and develop deeper understanding of community needs, gender roles, and other dynamics that facilitate successful uptake (Frangos et al. 2011; Kumar et al. 2016).

### Defining Success

Defining and measuring success is fundamental, but stove implementation programs too often use output measures, such as numbers of stoves or homes receiving distribution, rather than outcome-based measures of success. The long history of failed cooking interventions illustrates why this is insufficient. To improve health, households must use truly effective clean cooking technologies that displace more polluting technologies and sustain this use over time.

For household- and community-level measures, we propose two general categories of success measurement. The first focuses on household behavior—sustained predominant use of clean cooking systems; and the second focuses on resulting exposures—reductions in HAP exposure down to levels expected to yield health benefits based on identified exposure–response relationships in the absence of measured health outcomes. The latter is obviously of principal relevance to achieving the WHO targets and health objectives and fundamental to the research and technical evaluation of communities. The former is more likely to be of use to implementers, but even they must be cognizant of the causal chain and build in some level of exposure assessment for any program that aims to improve health outcomes.

The GACC recently released a framework of adoption and sustained use indicators that are intended to be widely used (GACC 2016). These are an important starting point for household, community and national level indicators for successful adoption, policy and coverage, promotion and uptake. The SE4All Global Tracking Framework offers another set of multi-tier measures focused on clean energy access (IEA 2015; see Annex pages 172–177). While sufficient empirical data to define adequate exposure reductions in most situations is still needed, the WHO IAQG and the published integrated exposure response (IER) curves (Burnett et al. 2014) provide critical guidance today for household and community targets.

For many policy makers, success will be defined by national health outcomes. When considering options for fuel, cookstoves, and program choices, the Household Air Pollution Intervention Tool (HAPIT) (Pillarisetti et al. 2016; see www.cleancookstoves.org/hapit) is a decision support tool that estimates potential health improvements and the relative cost-effectiveness of different fuel and cookstove policy and program strategies at a national level.

## Conclusions

Ambitious goals for scaling up clean cooking have been set by several international bodies, including the GACC, the South-East Asia Region of WHO, and the Sustainable Energy for All initiative of the United Nations Director General. Household energy will be included in several of the indicators to measure progress toward the Sustainable Development Goals (United Nations 2015). These targets are supported by governments, non-governmental organizations, donors, and companies around the world, to varying degrees, and efforts to distribute cleaner cookstoves and fuels are accelerating in dozens of countries. As both public and private initiatives around the globe begin to implement these interventions to reduce HAP exposure and its associated morbidity and mortality, we need to greatly improve our understanding of how to design and implement these interventions more effectively.

We believe that systematic study of access, adoption, and use of clean cooking technology that uses existing implementation science frameworks and that develops new synthetic approaches will provide powerful tools for understanding barriers to and enablers of adoption. The ISN can provide an important platform to synthesize lessons from around the world to share with both the implementation and research communities to inform policy and program and practice and to accelerate the intelligent and evidenced-based transition to cleaner cooking.
